# Simple Water Test

**Published:** 1900-06

**Authors:** 


### Simple Water Test.

Into a ground-glass, stoppered, perfectly clean bottle put five ounces of the water to be tested. To the water add ten grains of pure, granulated, white sugar. Cork tight and set in a window exposed freely to light, but not to direct rays of the sun. Do not disturb the bottle, and keep the temperature as near seventy degrees F. as possible. If the water contains organic matter, within forty-eight hours an abundance of whitish specks will be seen floating about, and the more organic matter the more specks. In a week or ten days, if the water is very bad, the odor of rancid butter will be noticed on removing the stopper. The little specks will settle to the bottom, where they appear as white flocculent masses. Such water should not be used for potable purposes.—*Iowa Health Bulletin.*

